# Genome-wide screening for the G-protein-coupled receptor (GPCR) pathway-related therapeutic gene *RGS19* (regulator of G protein signaling 19) in bladder cancer

**DOI:** 10.1080/21655979.2021.1971035

**Published:** 2021-09-05

**Authors:** Yue Liu, Weiming Lou, Guang Chen, Bing Ding, Jin Kuang, Yize Zhang, Cong Wang, Sainan Duan, Ying Deng, Xiongbing Lu

**Affiliations:** a Queen Mary School, Medical Collage of Nanchang University, Nanchang, China; b Institute of Translational Medicine, Nanchang University, Nanchang, China; c Department of Urology, The Second Affiliated Hospital of Nanchang University, Nanchang, China; d The Second Affiliated Hospital of Nanchang University, Nanchang University, Nanchang, Jiangxi Province, China; e The First Affiliated Hospital of Nanchang University, Nanchang University, Jiangxi Province, China; f The Second Affiliated Hospital of Nanchang University, Nanchang University, Jiangxi Province, China

**Keywords:** Bladder cancer, g-protein-coupled receptor, *rgs19*, gsk1070916, cell cycle

## Abstract

Bladder cancer is one of the most severe genitourinary cancers, causing high morbidity worldwide. However, the underlying molecular mechanism is not clear, and it is urgent to find target genes for treatment. G-protein-coupled receptors are currently a target of high interest for drug design. Thus, we aimed to identify a target gene-related to G-protein-coupled receptors for therapy. We used The Cancer Genome Atlas (TCGA) and DepMap databases to obtain the expression and clinical data of *RGS19*. The results showed that *RGS19* was overexpressed in a wide range of tumor, especially bladder cancer. We also explored its effect on various types of cancer. High expression of *RGS19* was also shown to be significantly associated with poor prognosis. Cell models were constructed for cell cycle detection. sh*RGS19* can halt the cell cycle at a polyploid point. *RGS19* is a G-protein-coupled receptor signaling pathway-related gene with a significant effect on survival. We chose *RGS19* as a therapeutic target gene in bladder cancer. The drug GSK1070916 was found to inhibit the effect of *RGS19* via cell rescue experiments *in vitro*.

## Introduction

Bladder cancer (BLCA) is one of the most severe genitourinary cancers. It is estimated that in 2018, approximately 550,000 people were diagnosed with BLCA, and it caused approximately 200,000 deaths [1,2]. Depending on the cellular infiltration of cancer cells in the muscular layer, the 8th edition of the AJCC manual classified BLCA into non-muscle-invasive bladder cancer (NMIBC) and muscle-invasive bladder cancer (MIBC). MIBC accounts for 25% of newly diagnosed cases, with a high propensity to metastasize and a 5-year survival rate of only 15% [3,4]. The treatment for BLCA usually depends on the type and stage. Radical cystectomy with pelvic lymph node dissections is considered the first-line treatment, and platinum-based combined chemotherapy is the essential protocol during the perioperative period to prolong survival [4]. Before surgery, cisplatin-based neoadjuvant chemotherapy may shrink the tumor and destroy cancer cells that are spreading to the surroundings [5,6]. Currently, the primary challenge is resistance to chemotherapeutic drugs, as studies demonstrate that more than 50% of MIBC therapy is not curative [7]. Adverse effects, including but not limited to mucositis, neutropenia, neutropenic fever, and neutropenic sepsis, are another problem for chemotherapy and cannot be overlooked [8]. Once metastasis occurs, there is no scheme to cure patients permanently. In this situation, a combined method of chemotherapy, immunotherapy and targeted therapy can prolong the life span [9]. In terms of targeted therapy, the FDA approved erdafitinib for patients who do not respond to chemotherapy. However, the short duration and toxicity to the eyes should be considered [10]. Two other targeted drugs, enfortumab vedotin-ejfv and sacituzumab govitecan, may also cause serious side effects in patients, such as anemia, and diarrhea [11]. To enhance therapeutic efficacy and reduce adverse effects, there is an urgent need for novel therapeutic targets.

As a type of seven-transmembrane proteins located on the cell surface, G-protein-coupled receptors (GPCRs) are the largest family of receptors in many organisms, including humans. Their main functions are detecting compounds on the cell surface and initiating several signaling cascades [12]. GPCRs are related to the pathogenesis of many diseases and are therefore widely used as targets for drug design. The vital role of GPCRs in multiple physiological functions and pathological mechanisms establishes them as targets for 34% of drugs approved by the Food and Drug Administration (FDA) [13]. GPCR-targeting drugs can act on multiple cancer-related processes, such as cell division, growth, differentiation, apoptosis, angiogenesis and microenvironment construction, which suggests great untapped potential in identifying new targets related to GPCRs and relevant pathways for new drugs to treat BLCA in the future [14]. However, there is no research focusing on GPCR-related pathways in BLCA therapy.

In this study, we aimed to identify a target gene involved in the GPCR pathway in BLCA and offer a novel treatment option for BLCA patients. We performed whole-genome screening *in silico* to investigate the genetic alterations associated with the G protein pathway in BLCA. We evaluated the abnormally high expression level and effects on the prognosis of *RGS19* in many types of cancer through integrated data analysis. Bioinformatic analysis and in vitro tests demonstrated the effect of *RGS19* on the cell cycle and the inhibitory effect of GSK1070916 on BLCA with abnormally high expression of *RGS19*. We hypothesized that *RGS19* is a potential therapeutic target for BLCA and that GSK1070916 is an alternative drug for its clinical treatment.

## Materials and methods

### Data acquisition and analysis

We downloaded the RNA-seq data of BLCA from The Cancer Genome Atlas (TCGA) database [15]. Based on the TCGA database, the gene expression of candidate genes and clinicopathological characteristics data were obtained from 16 types of cancer whose tumor and nontumor sample sizes were both more than 10. In the Gene Expression Omnibus (GEO) database (https://www.ncbi.nlm.nih.gov/geo/), we selected two profiles and downloaded the original (.CEL file) and platform files.

The DEGs between BLCA and normal samples were identified by the limma package [16]. The cutoff criteria were |log2 fold-change|>2 and false discovery rate (FDR) < 0.05. To identify potential biological pathways distinguishing low-risk and high-risk patients in the progression of BLCA, we analyzed the Kyoto Encyclopedia of Genes and Genomes (KEGG) pathways using Gene Set Enrichment Analysis (GSEA) [17]. FDR < 0.05 and p-value < 0.05 were deemed statistically significant. The GPCR signaling pathway data were downloaded from the Gene Ontology Annotation (GOA) database at the cBioPortal for Cancer Genomics (www.cbioportal.org).

## Cell culture

The BLCA cell line T24, SW780, 5637 and cell line SV-HUC-1, were purchased from the Cell Bank of the Type Culture Collection of the Chinese Academy of Sciences (Shanghai, China). The cells were cultured in Cell Counting Kit-8 (MedChemExpress, Shanghai, China) supplemented with 10% fetal bovine serum (Gibco, Gaithersburgh, MD, USA) and 100 U/mL penicillin–streptomycin (Beyotime Biotech, Beijing, China) in an incubator with 5% CO_2_ at 95% humidity and 37°C.

## RNA purification and quantitative RT-PCR

RNA was extracted using TRIzol reagent (Invitrogen). The complementary cDNA was transcribed using TaqMan Reverse Transcription Reagents (Applied Biosystems, Branchburg, New Jersey, USA). Real-time quantitative PCR (qRT-PCR) was performed using a two-step SYBR Green II fluorescent chimaeric real-time PCR system [18]. The sequence of the *RGS19* forward primer was 5ʹ-CCGTCTGACTT-GAGTCCCTG-3ʹ, and the sequence of the reverse primer was 5ʹ-CGTGGTACCAGCTCTCAGAC-3ʹ. *GAPDH* was used as an internal reference. The sequence of the *GAPDH* forward primer was 5ʹ-CCG-TCTGACTTGAGTCCCTG-3ʹ, and the sequence of the *GAPDH* reverse primer was 5ʹ-CGTGGTACC-AGCTCTCAGAC-3ʹ. The primers for cell cycle-related genes were indicated in Supplementary Table 1.

## Construction of shRNA and transfection

Oligonucleotides 5ʹ-CCGGGAGGCTCATCTAC-GAGGACTACTCGAGTAGTCCTCGTAGATGAGCCTCTTTTTG-3ʹ and 5ʹ-CCGGCCCTTCAA-TGTCCAGTCATGACTCGAGTCATGACTGGACATTGAAGGGTTTTTG-3ʹ were synthesized and annealed to produce a double-stranded shRNA template, which was amplified by PCR. Purified shRNA was digested with BglII and HindIII and inserted into pSUPER-retro-GFP/Neo. Constructs were verified by sequencing.

## Western blotting

Cells were lysed in RIPA buffer (Cell Signaling Technologies, Beverly, MA, USA). Proteins were separated on a 10% gel using sodium dodecyl sulfate polyacrylamide gel electrophoresis (SDS-PAGE). Protein was transferred to polyvinylidene difluoride membranes (Millipore, MA, USA). The membranes were immunoblotted with primary antibodies overnight at 4°C followed by the respective secondary antibodies. Primary antibodies against *RGS19* were obtained from Amylet Scientific (Wuhan, Hubei, China). The secondary antibodies were purchased from Santa Cruz Biotechnology (Dallas, TX, USA). *GAPDH* was used as a loading control. The relative expression was calculated as the ratio of drug-treated samples vs. control (DMSO) and corrected using the quantified level of *RGS19* expression.

## Cell cycle detection and flow cytometry analysis

BLCA cells were harvested, centrifuged and then washed twice with cold PBS. A DNA Content Quantitation Assay (Cell Cycle) (Solarbio, Beijing, China) was used to analyze the cell cycle process. The cell cycle distribution of each group of cells was analyzed by flow cytometry. Data were acquired with an Arial III flow cytometer (BD Bioscience) and analyzed with Tree Star FlowJo software.

## Colony formation assay

T24 cells (1,000/well) were cultured for 24 h in the presence or absence of germacrene. The medium was changed, and the culture continued until clear cell colonies were formed. Cells were then stained using the Wright–Giemsa Stain Kit (Nanjing Jiancheng Bioengineering Institute, Nanjing, Jiangsu, China). Transfected cells were harvested, washed twice with cold PBS, and fixed in cold 70% ethanol.

## Statistical analysis

The clinical data and expression data are expressed as the means ± SD. Differences between groups were estimated using the χ2-test or Student’s t-test. Overall survival and disease-free survival analyses were calculated according to the Kaplan–Meier method with the log-rank test to examine the differences in the incidence of death between the high-*RGS19* group and the low-*RGS19* group. A Cox regression analysis (proportional hazards model) was performed for the multivariate analyses of prognostic factors. All analyses were conducted using SPSS 16.0 software (IBM, Armonk, NY, USA), and significance was defined as a two-tailed value of P < 0.05.

## Results

In this study, we sought to identify a gene related to the GPCR signaling pathway in BLCA as a potential treatment target. Based on the data from the TCGA and DepMap databases, comprehensive analysis proved significantly high expression of *RGS19* in BLCA, which was related to tumor progression and poor prognosis. The influence of *RGS19* expression on cancer pathogenesis was explored, and pathway analysis and cell models verified its function in the cell cycle. GSK1070916 may serve as a potential drug to treat abnormally high expression of *RGS19* in BLCA.

## Identification of *RGS19* from GPCR-related genes

**Figure 1.**
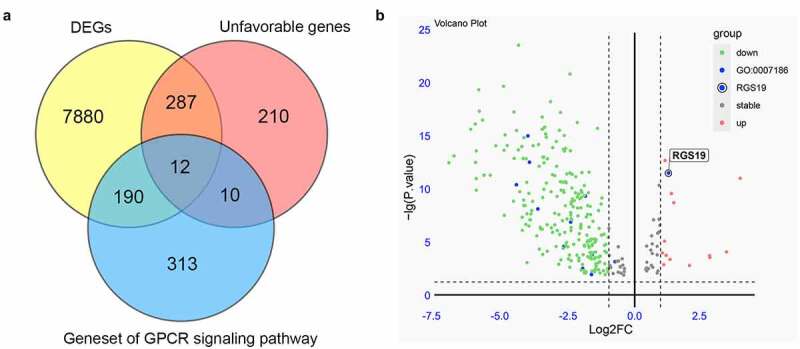
**Screening essential GPCR-related genes for BLCA**. (a) Through the integration of differential expression analysis, pathway analysis and survival analysis, we identified 12 essential GPCR-related genes that play an important role in BLCA. (b) The volcano plot was constructed based on differential expression analysis. The expression of *RGS19* in BLCA was significantly upregulated (P = 4.2x10^−13^)

We collected RNA-seq data from the TCGA database to screen for essential GPCR-related genes in BLCA. Whole-genome data and ten-year survival data were downloaded, and differential expression analysis and survival analysis were performed. Finally, 8,369 DEGs were identified from 411 BLCA samples and 19 nontumor samples. In addition, a survival analysis of BLCA-related genes was performed, and 519 unfavorable genes were identified whose overexpression was related to poor prognosis. As mentioned above, due to the importance of the GPCR pathway in drug design, we finally collected a gene set of the GPCR signaling pathway containing 525 genes. We overlapped the above three gene groups and obtained 12 genes, including *ADCYAP1R1, AGTR1, AVPR1A, PTGER3, S1PR1, ARHGEF17, ADRA2A, ADRA1D, MC4R, ADCY5, RGS19*, and *DRD2* (Figure 1a). Only *RGS19* was significantly overexpressed among these 12 essential GPCR-related genes (P = 4.2x10^−13^, Figure 1b). Thus, we concluded that *RGS19* plays a very important role in BLCA and set it as our target.

## The effects of *RGS19* in many types of cancer and survival analysis

**Figure 2.**
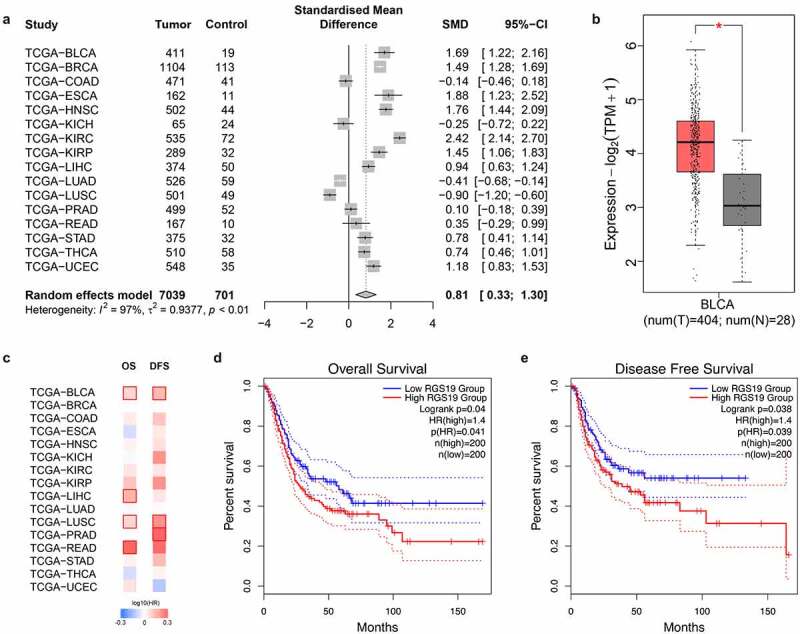
**Overview of the expression levels of *RGS19* between tumor and nontumor samples and summary of clinical analysis based on the TCGA**. (a) The forest plot of the expression levels among 16 types of tumor, including breast invasive carcinoma (BRCA), colon adenocarcinoma (COAD), esophageal carcinoma (ESCA), head and neck squamous cell carcinoma (HNSC), kidney chromophobe carcinoma (KICH), kidney renal clear cell carcinoma (KIRC), kidney renal papillary cell carcinoma (KIRP), liver hepatocellular carcinoma (LIHC), lung adenocarcinoma (LUAD), lung squamous cell carcinoma (LUSC), prostate adenocarcinoma (PRAD), rectum adenocarcinoma (READ), stomach adenocarcinoma (STAD), thyroid carcinoma (THCA), and uterine corpus endometrial carcinoma (UCEC). (P < 0.01). (b) We analyzed the expression levels of *RGS19* in 404 BLCA tumor samples and 28 nontumor samples. The box plot showed that *RGS19* was highly expressed in BLCA tissue (P < 0.01). (c) The heatmap of the expression levels. (d) Overall survival analysis showed that the group of high-*RGS19* patients had a worse prognosis (P = 0.041). (e) Disease-free survival analysis revealed that the survival time of high-*RGS19* patients was shorter than that of low-*RGS19* patients (P = 0.039)

We aimed to explore the effects and prognosis of *RGS19* in various types of tumor. Therefore, we collected expression data on 7,740 samples from 16 types of tumor and 701 corresponding nontumor samples. The expression of *RGS19* was high in most types of tumor (P < 0.01, Figure 2a), including BLCA (P < 0.01, Figure 2b). To further confirm the abnormally high expression of *RGS19* in BLCA, we evaluated the expression levels of *RGS19* in BLCA cell lines T24, SW780 and 5637 b, compared with those in normal human bladder cells, SV-HUC-1 cell line. The qRT-PCR results presented a consistent trend with bioinformatic analysis (Supplementary Figure 1).

We also performed survival analysis of *RGS19* in these 16 types of tumor, and the results for BLCA showed significant association with both OS and DFS (P < 0.05, Figure 2c). According to the median *RGS19* expression level, the patients were divided into high- and low-*RGS19* groups to draw a Kaplan–Meier curve. High *RGS19* expression was negatively correlated with OS (P = 0.041, Figure 2d) and DFS (P = 0.039, Figure 2e). The data of TCGA_BLCA, GSE32894 and GSE31684 were used for meta-analysis and survival analysis to validate the prognostic value of RGS19. The forest plot showed that *RGS19* was a steadily unfavorable factor (I^2^ = 7%, t^2^ = 0.0010, p = 0.34, Supplementary Figure 2). The nomogram plot was built based on four independent prognostic factors in BLCA. The results showed that *RGS19* could predict survival more accurately combined with the TNM stage (Supplementary Figure 3).

## The effect of *RGS19* on cell proliferation and clone formation

**Figure 3.**
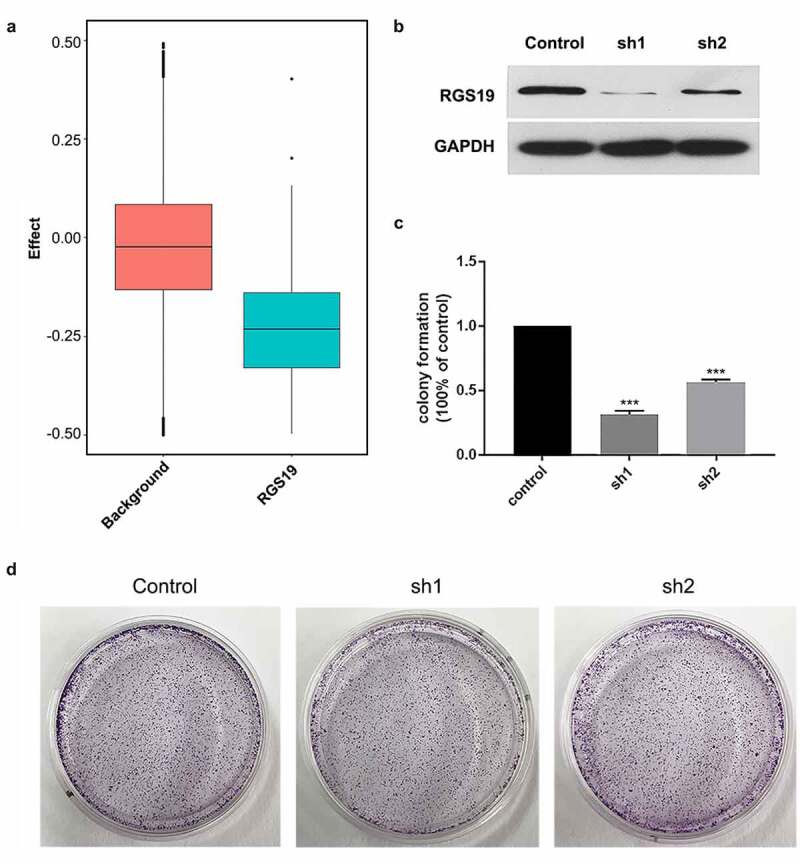
**Colony formation experiments with sh*RGS19***. (a) Compared to the background group, the knockout effect of *RGS19* in 1,000 cell lines was significantly stronger. (b) The Western blot results of two constructed shRNA plasmids. sh*RGS19*-1 showed a more significant effect on *RGS19* silencing. (c and d). After the knockdown, the number of colonies was decreased significantly. The effect of sh*RGS19*-1 was more potent

Cell models were established to study the effects of *RGS19* in BLCA. Based on the DepMap database, we evaluated the knockout effect of RGS19 in 1,000 cell lines. The background group consisted of 1,000 random knockout genes in 1,000 cell lines. The results showed that the survival ratio of RGS19 knockout was significantly lower (Figure 3a). This result indicated that *RGS19* was an essential gene for cells.

Cell models were established to study the effects of *RGS19* in BLCA. We conducted *in vitro* experiments on T24 cell lines to explore the silencing effect of sh*RGS19* on BLCA. We constructed two shRNA plasmids and detected their silencing effects using Western blotting (Figure 3b), which revealed that sh*RGS19*-1 had a better effect (Figure 3c). The two shRNA plasmids were also introduced separately into the T24 cell line, and then clone formation of the cells was observed. The results showed that sh*RGS19*-1 had a significant inhibitory effect on the number of clones (Figure 3d).

## The molecular mechanism of *RGS19* related to the cell cycle

**Figure 4.**
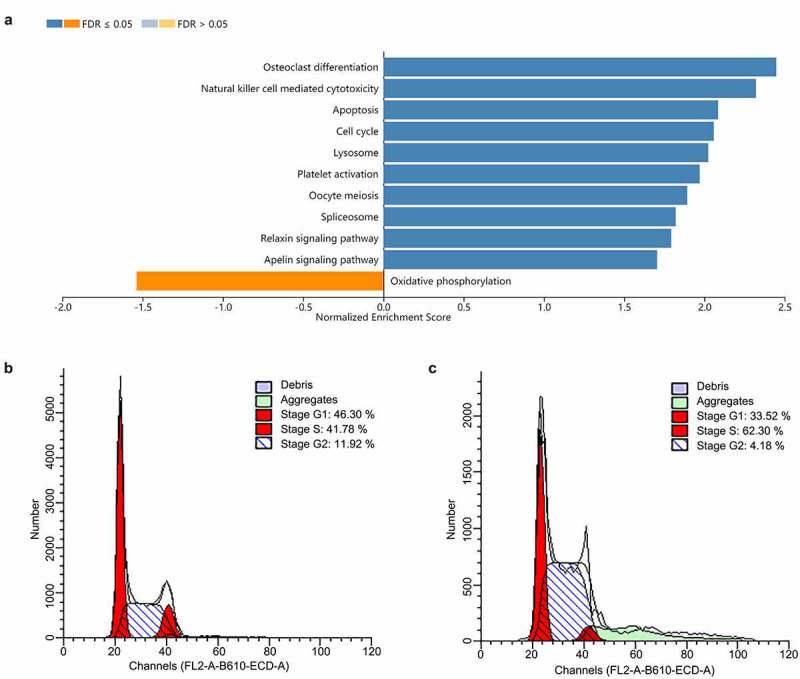
***RGS19* regulated the cell cycle pathway**. (a) GSEA of the transcriptome in BLCA tissue suggested that *RGS19* is related to the activation of the cell cycle pathway. (b and c) According to the results of cell cycle analysis, sh*RGS19* caused cell cycle disorder with polyploids in T24 cells

**Figure 5.**
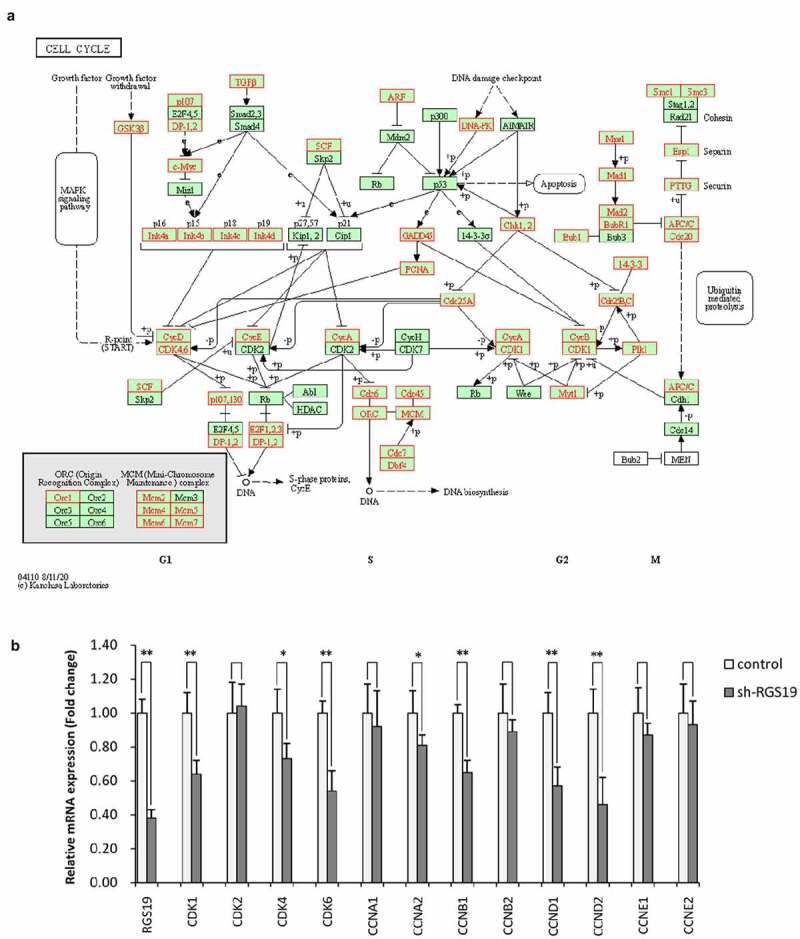
**The expression of genes involved in the cell cycle pathway was associated with *RGS19.*** (a) Genes involved in cell cycle pathway. (b) The results of qRT-PCR showed that sh-*RGS19* caused the lower expression of 8 key genes in the cell cycle

To further explore the biological significance of *RGS19* in BLCA, we performed pathway analysis based on the RNA-seq data of BLCA. We found that it was related to the activation of the cell cycle based on GSEA (Figure 4a). We also constructed cell models to explore the effect of *RGS19* on the cell cycle. The results of cell cycle analysis showed that the cell cycle of T24 cells was disordered, with more polyploid cells appearing (Figure 4b and 4c). Under normal conditions, polyploidy will trigger apoptosis [19,20]. However, apoptosis was inhibited because of kinase inhibition at this checkpoint. Therefore, after *RGS19* knockdown, a large number of BLCA cells will be stalled as polyploids. In addition, the results of qPCR showed that *RGS19* promoted the expression of 8 important genes that participated in the cell cycle pathway according to the data from the KEGG database (Figure 5a and 5b).

## Drug screening for BLCA treatment

**Figure 6.**
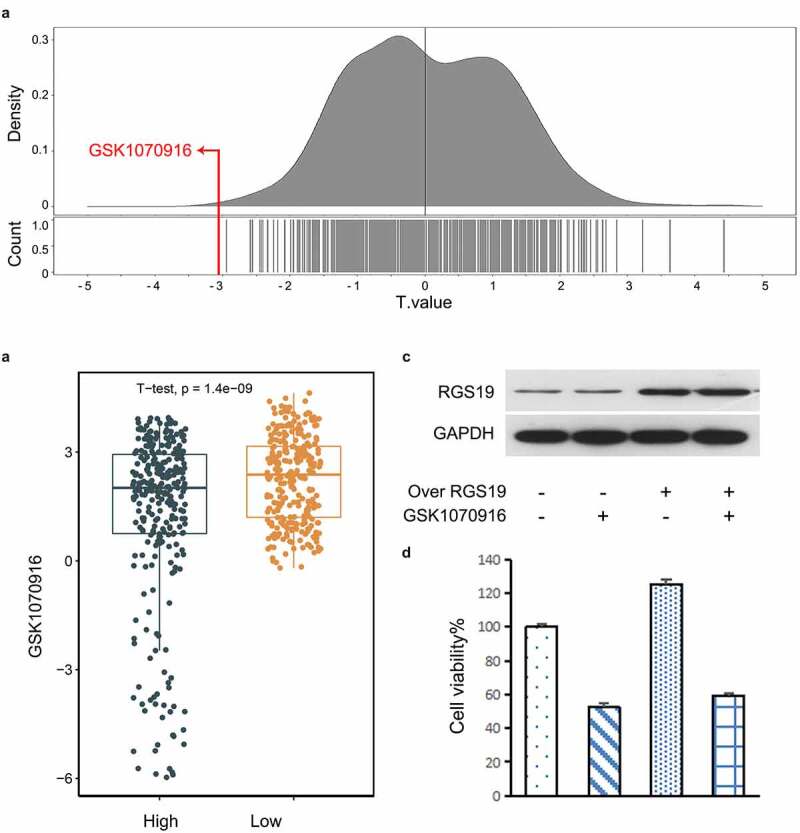
**Drug screening for targeting *RGS19.*** (a) In the GDSC database, we found that GSK1070916 was the most significant with the smallest t value. The negative t value indicated that overexpression of this gene was highly sensitive to this drug. (b) Targeted drug screening for *RGS19* based on IC50. T24 cells were most sensitive to GSK1070916, with the smallest t value (P = 1.4 x 10^−9^). (c and d) The western blot and cell proliferation results of the inhibitory effect of GSK1070916 on *RGS19* overexpression

Based on the Genomics of Drug Sensitivity in Cancer (GDSC) database, we explored possible drugs that might affect *RGS19*. We used the 50% inhibitory concentration (IC50) as the standard for screening. The results showed that BLCA cells with high expression of *RGS19* were most sensitive to GSK1070916, with the smallest t value based on the IC50 (Figure 6a). We used the median *RGS19* expression level as the standard and divided the cell lines into ‘High’ and ‘Low’ groups. The t value in the ‘High’ group was significantly lower than that in the ‘Low’ group (P = 1.4 x 10^−9^, Figure 6b).

To further verify the inhibitory effect of GSK1070916 on *RGS19*, we conducted a cellular experiment. We induced *RGS19* overexpression in T24 cells. The downregulation of *RGS19* caused by GSK1070916 was abrogated at both the protein and mRNA levels (Figure 6c and 6d). We analyzed the cell proliferation rate and found that it was increased in *RGS19*°^ver+^GSK1070916^−^ T24 cells, but this effect was abrogated in RGS19°^ver+^GSK1070916^+^ T24 cells (Figure 6d), suggesting that GSK1070916 can inhibit T24 cell proliferation by silencing the overexpression of *RGS19*.

## Discussion

The value and novelty of our study was the discovery that *RGS19* is a critical gene in BLCA through genome-wide screening, cell experiments and drug screening in cell lines with high *RGS19* expression. There were three key points and main aims in our research. We first performed screening and identified a GPCR-related gene, *RGS19*, as a candidate for therapeutic targeting of BLCA. Then, we systematically showed the importance of *RGS19* for the oncogenesis of BLCA at different levels by comprehensive multi-omics analysis. Moreover, we demonstrated the association of *RGS19* with the cell cycle and the inhibitory effect of GSK1070916 on cells with high *RGS19* expression.

*RGS19* (regulator of G protein signaling 19) belongs to the regulators of the G-protein signaling (RGS) family. RGS proteins are negative regulators of GPCR signaling through their ability to act as GTPase-activating proteins (GAPs) for activated Gα subunits [21]. The RGS domain modulates signaling pathways initiated by GPCRs in a way that facilitates the deactivation of heterotrimeric G-proteins [22]. Previous studies have shown that RGS proteins participate in diverse cellular activities, including cell proliferation, differentiation, and apoptosis [23]. In addition to a conserved RGS domain responsible for GAP activities, different RGS proteins have different signaling motifs [24]. RGS19 has a C-terminal PDZ-binding motif used to interact with GIPC, which in turn associates RGS19 with downstream signals [25]. A GIPN, E3 ubiquitin ligase, binds to the N-terminus to induce the degradation of Gia through a ubiquitin proteasome-dependent pathway [26]. In addition, RGS19 has been associated with the carcinogenesis of several other cancers, including ovarian cancer, primary kidney tumor, gastric cancer, prostate cancer and colorectal tumor [22,27,28]. In our study, we first confirmed the overexpression and adverse effects of *RGS19* in BLCA. Next, our bioinformatic analysis showed that upregulation occurred in the pancancer dataset and was associated with poor prognosis as an unfavorable factor. Among multiple cancers, high expression of *RGS19* showed a particularly strong impact on BLCA, because both OS and DFS were significantly lower in BLCA patients with high RGS19 expression than in those with low RGS19 expression. Pancancer analysis reinforced the importance of *RGS19* in BLCA. All these facts suggest that *RGS19* is a potential therapeutic target in BLCA. It is reported that the expression of CD44 is lower in BLCA than in none tumor tissue and it is associated with TNM staging [29]. Another study found that TERT promoter mutations may predict BLCA recurrence and become a novel target for BLCA treatment [30]. Previous studies had explored the associations between RGS family members and bladder cancer risk, including *RGS1, RGS2, RGS4, RGS5, RGS6*, and *RGS20*. Our study is the first research that identified the risk of *RGS19* on BLCA by means of bioinformatic analysis in combination with experiment. In addition, we found a potential therapeutic drug, GSK1070916, for BLCA patients with high expression of RGS19 [31–34].

In this report, pathway analysis showed the involvement of RGS19 in the cell cycle. Furthermore, subsequent cellular experiments supported this idea. After RGS19 knockdown, cell cycle arrest led to polyploid cells. Previous research in non-small cell lung carcinoma (NSCLC) also supported the role of RGS19 in the cell cycle, as the suppression of tumorigenesis after knocking down RGS19 cell line H1299 suggested that RGS19 can facilitate the process of oncogenesis [35]. However, the mechanism by which RGS19 dysregulates the cell cycle in BLCA is not fully understood. Past work has pointed out that RGS19 promotes the cell cycle by upregulating cyclin D1/3 and cyclin-dependent kinase 6, the molecules responsible for the G1/S transition, through the phosphatidylinositol 3ʹ-kinase (PI3K)-Akt pathway, and this mechanism is independent of its role as a GAP [25,36].

In our study, we found that GSK1070916, an inhibitor of Aurora kinase B/C, significantly suppressed BLCA when the expression level of *RGS19* was upregulated. An inhibitor of Aurora kinase B was reported to disturb normal chromosome segregation and resulted in the appearance of polyploids [37]. Aurora B is highly expressed in various cancers, including NSCLC [38]. As mentioned above, *RGS19* knockdown interfered with the cell cycle in NSCLC. These two facts together suggested a possible mechanism involving the RGS19-AURKB pathway. In the cell cycle assay, after *RGS19* knockdown, BLCA cells transformed into polyploids. AURKB can upregulate the expression of *CCND1*, which encodes cyclin D1 [39]. All of these clues suggest the novel conclusion that RGS19 regulates AURKB through the Akt-PI3K pathway.

GSK1070916 is a reversible and ATP-competitive inhibitor of Aurora B/C, which is encoded by an essential gene controlling multiple events in the cell cycle and mitosis [40]. Its inhibition of proliferation has a broad-spectrum effect on more than 100 cell lines from multiple tumor types [41]. Some previous studies confirmed the anti-tumor effect of GSK1070916 in human tumor xenograft models, including colon, breast, lung and leukemia [41,42]. The Aurora kinase family, which was initially identified in Drosophila, consists of Aurora kinase A, Aurora kinase B and Aurora kinase C [43]. The main functions of Aurora kinase are linked to the regulation of cellular mitosis [44]. Aurora kinase B is a chromosomal passenger protein that forms a chromosomal passenger protein complex (CPC) with three other chromosomal passenger proteins, inner-centromere protein (INCENP), borealin, and survin. In the early stage of mitosis, Aurora B is distributed along the chromosome arm and then aggregates on the centromere of the chromosome and remains until the middle of the division. The complex helps Aurora B instigate spatial displacement [45–47]. The function of CPC is mostly regulated by Aurora kinase B, whereas Aurora C requires further exploration. Therefore, we focused on the regulation of Aurora kinase B [44]. We infer that RGS19 may influence the expression of Aurora kinase B or its partner proteins, including INCENP, borealin, and survin. A number of studies have demonstrated that Aurora kinase is highly expressed in many tumor tissues. However, it is activated only during mitosis, and its expression in nonproliferating cells is low. In the human body, most normal cells do not proliferate at a rapid rate. Therefore, inhibitors targeting Aurora kinase have an advantage than nonspecific drugs cannot match.

Based on the existing literature and experimental results, we propose the following assumptions. The *RGS19*-AURKB pathway is a pivotal process in the regulation of the BLCA phenotype, and *RGS19* is a promising new target for targeted BLCA therapy. We hypothesized that *RGS19* regulates AURKB through the PI3K-Akt pathway. However, this hypothesis requires further exploration and verification. *RGS19* is a potential biomarker for predicting the effect of AURKB/C inhibitors in BLCA therapy. Finally, several limitations to this study should be considered. We did not investigate the concrete molecular mechanism of how the *RGS19*-AURKB/C pathway influences the phenotype of BLCA cells in clinical trials or in vitro models.

## Conclusion

The upregulated expression of *RGS19* showed a significant association with poor prognosis in BLCA. *RGS19* was found to participate in cell cycle regulation. GSK1070916, an inhibitor of Aurora kinase B/C, inhibited the proliferation of BLCA with high *RGS19* expression. Therefore, *RGS19* might serve as a promising therapeutic target for BLCA, and GSK1070916 has potential as a BCLA treatment option.

## Supplementary Material

Supplemental Material

## Highlights

(1) *RGS19* is a potential therapeutic target gene involved in the GPCR pathway in BLCA.

(2) *RGS19* is associated with the regulation of the cell cycle.

(3) GSK1070916 was proven to be a promising antitumor drug in BLCA treatment.
