# Supplementary figures and images for: Genome-wide screening for the G-protein-coupled receptor (GPCR) pathway-related therapeutic gene RGS19 (regulator of G protein signaling 19) in bladder cancer
Source: Bioengineered. 2021 Sep 5;12(1):5892–903. doi: 10.1080/21655979.2021.1971035 (PMC8806424; doi:10.1080/21655979.2021.1971035)

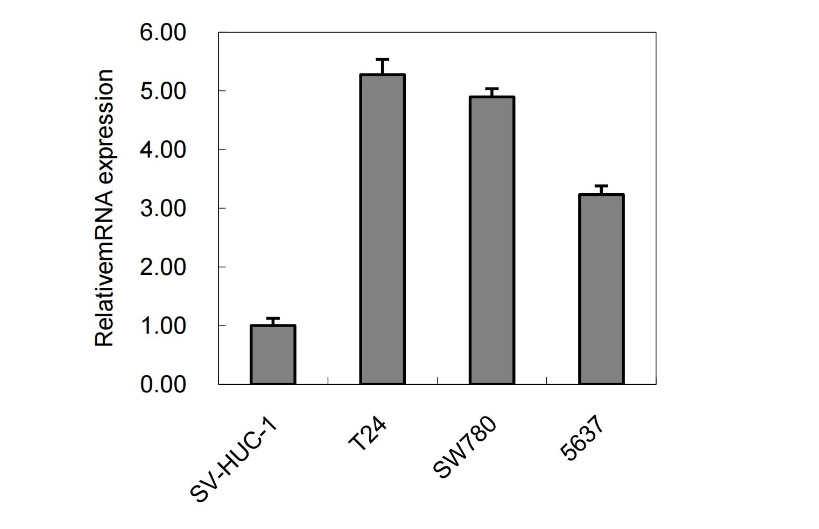

Supplement: Supplemental Material [file KBIE_A_1971035_SM7354.zip › supplementary/Supplementary Figure 1.jpg]

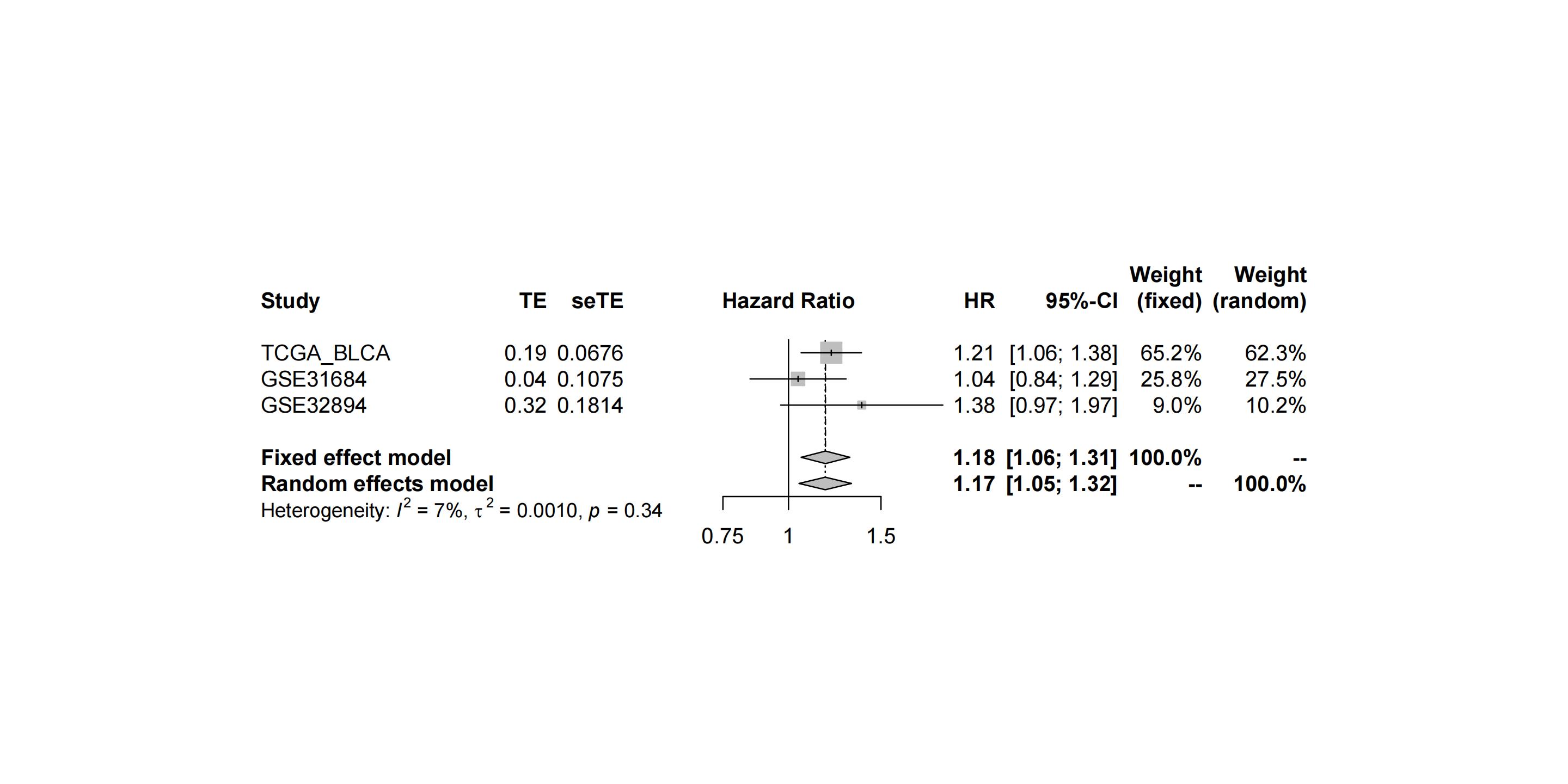

Supplement: Supplemental Material [file KBIE_A_1971035_SM7354.zip › supplementary/Supplementary Figure 2.jpg]

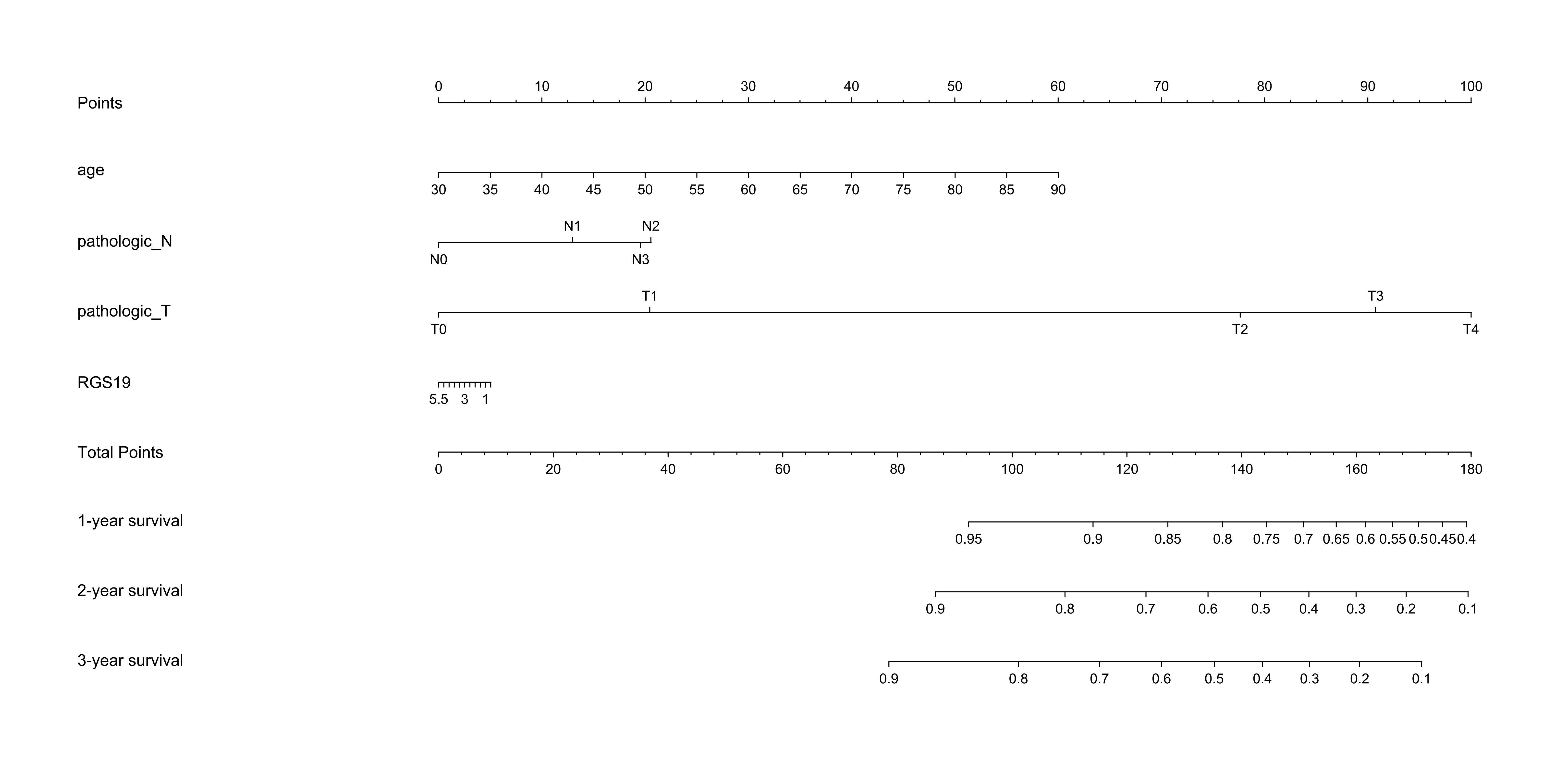

Supplement: Supplemental Material [file KBIE_A_1971035_SM7354.zip › supplementary/Supplementary Figure 3.jpeg]
