# Supplementary material for: Genome-wide screening for the G-protein-coupled receptor (GPCR) pathway-related therapeutic gene RGS19 (regulator of G protein signaling 19) in bladder cancer
Source: Bioengineered. 2021 Sep 5;12(1):5892–903. doi: 10.1080/21655979.2021.1971035 (PMC8806424; doi:10.1080/21655979.2021.1971035)
Supplement: Supplemental Material [file KBIE_A_1971035_SM7354.zip › supplementary/Supplementary Table 1.doc]

**Supplementary Table 1: The primers of Quantitative RT-PCR**.

| **Gene list** | **Sequence (5’-3’)** |
| --- | --- |
|  |  |
| CDK1 Forward Primer | AAACTACAGGTCAAGTGGTAGCC |
| CDK1 Reverse Primer | TCCTGCATAAGCACATCCTGA |
| CDK2 Forward Primer | CCAGGAGTTACTTCTATGCCTGA |
| CDK2 Reverse Primer | TTCATCCAGGGGAGGTACAAC |
| CDK4 Forward Primer | ATGGCTGCCACTCGATATGAA |
| CDK4 Reverse Primer | TCCTCCATTAGGAACTCTCACAC |
| CDK6 Forward Primer | TCTTCATTCACACCGAGTAGTGC |
| CDK6 Reverse Primer | TGAGGTTAGAGCCATCTGGAAA |
| CCNA1 Forward Primer | GCCTGGCAAACTATACTGTG |
| CCNA1 Reverse Primer | CTCCATGAGGGACACACACA |
| CCNA2 Forward Primer | GGTACTGAAGTCCGGGAACC |
| CCNA2 Reverse Primer | GAAGATCCTTAAGGGGTGCAA |
| CCNB1 Forward Primer | CATGGTGCACTTTCCTCCTT |
| CCNB1 Reverse Primer | AGGTAATGTTGTAGAGTTGGTGTCC |
| CCNB2 Forward Primer | CCGACGGTGTCCAGTGATTT |
| CCNB2 Reverse Primer | TGTTGTTTTGGTGGGTTGAACT |
| CCND1 Forward Primer | GCTGCGAAGTGGAAACCATC |
| CCND1 Reverse Primer | CCTCCTTCTGCACACATTTGAA |
| CCND2 Forward Primer | ACCTTCCGCAGTGCTCCTA |
| CCND2 Reverse Primer | CCCAGCCAAGAAACGGTCC |
| CCNE1 Forward Primer | AAGGAGCGGGACACCATGA |
| CCNE1 Reverse Primer | ACGGTCACGTTTGCCTTCC |
| CCNE2 Forward Primer | TCAAGACGAAGTAGCCGTTTAC |
| CCNE2 Reverse Primer | TGACATCCTGGGTAGTTTTCCTC |
